# Supplementary material for: Diagnosing missed cases of spinal muscular atrophy in genome, exome, and panel sequencing datasets
Source: medRxiv. 2024 Jun 29:2024.02.11.24302646. Originally published 2024 Feb 13. Preprint. [Version 3] doi: 10.1101/2024.02.11.24302646 (PMC10889006; doi:10.1101/2024.02.11.24302646)
Supplement: 1 [file NIHPP2024.02.11.24302646V3-supplement-1.pdf]

medRxiv preprint doi: https://doi.org/10.1101/2024.02.11.24302646; this version posted June 29, 2024. The copyright holder for this preprint (which was not certified by peer review) is the author/funder, who has granted medRxiv a license to display the preprint in perpetuity.
It is made available under a CC-BY 4.0 International license .

Supplementary Materials

Installing and Running SMA Finder

The SMA Finder tool is implemented in python v3.7+ and can be installed by running

```
python3 -m pip install sma_finder
```

The --help option can be used to print out SMA Finder's command line arguments:

```
python3 -m sma_finder --help

positional arguments:
  cram_or_bam_path       One or more CRAM or BAM file paths

optional arguments:
  -h, --help             show this help message and exit
  --hg37-reference-fasta HG37_REFERENCE_FASTA
                         HG37 reference genome FASTA path. This should
be specified if the input bam or cram is aligned to HG37.
  --hg38-reference-fasta HG38_REFERENCE_FASTA
                         HG38 reference genome FASTA path. This should
be specified if the input bam or cram is aligned to HG38.
  --t2t-reference-fasta T2T_REFERENCE_FASTA
                         T2T reference genome FASTA path. This should
be specified if the input bam or cram is aligned to the CHM13
telomere-to-telomere benchmark.
  -o OUTPUT_TSV, --output-tsv OUTPUT_TSV
                         Optional output tsv file path
  -v, --verbose          Whether to print extra details during the run
```

SMA Finder has two required inputs:

1) a reference genome file provided via the appropriate `--*-reference-fasta` option
2) the path(s) of one or more aligned read data files in bam or cram format

It outputs a tab-separated table with one row per input file with the following columns:

filename_prefix = input read data filename without the “.bam” or “.cram” suffix
file_type = “bam” or “cram”

medRxiv preprint doi: https://doi.org/10.1101/2024.02.11.24302646; this version posted June 29, 2024. The copyright holder for this preprint (which was not certified by peer review) is the author/funder, who has granted medRxiv a license to display the preprint in perpetuity.
It is made available under a CC-BY 4.0 International license .

genome_version = "hg37", "hg38", or "t2t"
sample_id = sample id parsed from the read data file header
sma_status = "does not have SMA", "has SMA", or "not enough coverage at SMN c.840 position"
confidence_score = estimates the degree of confidence in the "sma_status" value
c840_reads_with_smn1_base_C = number of reads with a "C" nucleotide at the c.840 position in *SMN1* + *SMN2*
c840_total_reads = total number of reads that overlap the c.840 position in *SMN1* + *SMN2*

Table S1. Example SMA Finder output table

filename_prefix	file_type	genome_version	sample_id	sma_status	confidence_score	c840_reads_with_smn1_base_C	c840_total_reac
sample1	bam	hg38	s5	does not have SMA	2524	85	146
sample2	cram	hg38	MWAR098	has SMA	182	1	218
sample3	cram	hg37	s12421	not enough coverage at SMN c.840 position	0	2	8

Table S2. Concordance between SMA Finder exome calls vs SMNCopyNumberCaller genome calls in 198,868 UKBB participants

```
198,818 individuals      negative call by both SMA Finder & SMNCopyNumberCaller
      1 individual (i1)  positive call by both SMA Finder & SMNCopyNumberCaller
      1 individual (i2)  positive call by SMA Finder, no-call by SMNCopyNumberCaller
     34 individuals      negative call by SMA Finder, no-call by SMNCopyNumberCaller
     14 individuals            no-call by SMA Finder, negative call by SMNCopyNumberCaller
```

Table S3. Concordance between SMA Finder calls in UKBB exomes vs genomes

```
198,772 individuals           negative call for both their exome and genome
      2 individuals (i1, i2)  positive call for both their exome and genome
      5 individuals           no-call for both their exome and genome
     80 individuals           negative call for their exome, no-call for their genome
      9 individuals           no-call for their exome, negative call for their genome
```

medRxiv preprint doi: https://doi.org/10.1101/2024.02.11.24302646; this version posted June 29, 2024. The copyright holder for this preprint (which was not certified by peer review) is the author/funder, who has granted medRxiv a license to display the preprint in perpetuity.
It is made available under a CC-BY 4.0 International license .

SMN sequences within GRCh38 ALT contigs

In addition to the standard *SMN1* and *SMN2* gene annotations on chr5, the GRCh38 reference contains three more copies of SMN within its ALT contigs. Specifically, a copy of *SMN1* can be found at chr5_KI270897v1_alt:473491-501447, a copy of *SMN2* occurs at chr5_KI270897v1_alt:274951-303848, and a reverse complement sequence of *SMN1* occurs at chr5_GL339449v2_alt:456849-485731. Hypothetically, during read alignment, these extra copies could absorb informative reads and distort the chr5 read counts that SMA Finder relies on to distinguish positive from negative samples. In practice, the BWA MEM aligner performs ALT-aware alignment so that, if a read has comparable alignments to both chromosomal and ALT contig sequence(s), BWA will report the chromosome alignment as the primary one, while any ALT contig alignments will be marked as secondary [†]. Since SMA Finder does not count secondary alignments, we assumed that it would be safe to ignore these extra SMN sequences on ALT contigs. To test the validity of this assumption, we reanalyzed all exome and genome samples within the CMG cohort to confirm that there were no primary alignments overlapping the c.840 positions of these three extra SMN copies on ALT contigs. Specifically, we used a modified version SMA Finder to count reads at the following genomic positions which correspond to c.840 of the three alternative SMN sequences: chr5_KI270897v1_alt:500378 (*SMN1*), chr5_KI270897v1_alt:301867 (*SMN2*), and chr5_GL339449v2_alt:458845 (reverse complement *SMN1*). As expected, in all CMG samples there were zero primary alignments overlapping these three positions, confirming that SMA Finder could ignore the GRCh38 ALT contigs as long as its input read data was aligned using BWA MEM or a functionally-equivalent aligner.

† See https://github.com/lh3/bwa/blob/master/README-alt.md#step-1-bwa-mem-mapping for a detailed description of BWA's approach to ALT contigs.

Detailed description of the SMA Finder algorithm

SMA Finder starts by retrieving aligned reads that overlap the c.840 position in *SMN1* and *SMN2*. Then, it computes two read counts, **r** and **N**, which are defined as follows:

N = total number of reads that overlap the c.840 position in *SMN1* + *SMN2*
r = the number of reads that have a 'C' base at the c.840 position of *SMN1* or *SMN2* and therefore support the presence of at least one intact copy of *SMN1*

These counts include reads whose mapping quality equals zero, but exclude reads whose base quality score at the c.840 position is < 13.

medRxiv preprint doi: https://doi.org/10.1101/2024.02.11.24302646; this version posted June 29, 2024. The copyright holder for this preprint (which was not certified by peer review) is the author/funder, who has granted medRxiv a license to display the preprint in perpetuity.
It is made available under a CC-BY 4.0 International license .

When **N** ≥ 14, SMA Finder uses maximum likelihood estimation to determine whether, given these counts, it is more likely that the sample has zero functional copies of *SMN1*, or that it has more than zero copies. Otherwise, if **N** < 14, SMA Finder reports that the sample has insufficient read coverage to make a call.

When computing likelihoods, SMA Finder makes the following assumptions:

Assumption 1: The probability that a sequencing error caused a particular base within a given read to be incorrectly called a 'T' when the true base was a 'C' is = 0.001 / 3. This is similar to calculations used within GATK HaplotypeCaller's reference confidence model.[7] We do not rely on base quality scores for this parameter since their accuracy can depend on the sequencing technology used and whether algorithms like GATK Base Quality Score Recalibration were applied prior to running SMA Finder. Instead, we uniformly set **p_error = 3.3e-4**.

Assumption 2: Let's say **SMN1_cn** and **SMN2_cn** represent the copy numbers of *SMN1* and *SMN2* in a given sample. If an individual has at least one functional copy of *SMN1*, then the probability of the observed read counts **r** and **N** can be modeled by the binomial distribution B(**r, N, p)** where **p** = **SMN1_cn** / (**SMN1_cn** + **SMN2_cn**). On the other hand, if an individual has zero intact *SMN1* copies due to a mutation disrupting the *SMN1* c.840 position, any observed reads with a C at that position are assumed to be sequencing errors, and so the read counts are instead modeled by B(**r, N, p_error**)**.**

Assumption 3: We assume that, in any given sample, the true value of **SMN1_cn** + **SMN2_cn** **≤ 5** based on the data in Chen et al.[3] which found that genomes rarely contain more than 5 total copies of these paralogs.

Given these assumptions, the algorithm computes the likelihood of observing counts **r** and **N** in each of six scenarios: **SMN1_cn = 0, 1, 2, 3, 4,** or **5**, while keeping **SMN1_cn + SMN2_cn = 5**. Then, it concludes that a sample has zero functional copies of *SMN1* if the likelihood for **SMN1_cn** = 0 is greater than the likelihood for any other value of **SMN1_cn** between 1 and 5.

Since we don't know the true value of **SMN1_cn + CMN2_cn** in a given sample, we always set **SMN1_cn + SMN2_cn** to 5 when computing likelihoods because the resulting model is the least likely to produce a false positive result compared to if we had chosen a smaller value for **SMN1_cn + SMN2_cn**.

SMA Finder also computes a Phred-scaled confidence score by subtracting the log likelihood for **SMN1_cn** = 0 from the maximum log likelihood for **SMN1_cn** = i when i is between 1 and 5:

confidence score = 10 * abs(log10(B(r, N | **SMN1_cn = 0**)) - log10(max({ B(r, N | **SMN1_cn = i**) : i = 1, 2, 3, 4, 5 }))).

medRxiv preprint doi: https://doi.org/10.1101/2024.02.11.24302646; this version posted June 29, 2024. The copyright holder for this preprint (which was not certified by peer review) is the author/funder, who has granted medRxiv a license to display the preprint in perpetuity.
It is made available under a CC-BY 4.0 International license .
